# CAR-T免疫疗法与非小细胞肺癌：瓶颈与曙光

**DOI:** 10.3779/j.issn.1009-3419.2020.103.10

**Published:** 2020-10-20

**Authors:** 立 张, 恒 李, 飞越 张, 书廷 王, 高峰 李

**Affiliations:** 650118 昆明，云南省肿瘤医院胸外科 Department of Thoracic Surgery, Yunnan Cancer Hospital, Kunming 650118, China

**Keywords:** 肺肿瘤, 嵌合抗原受体修饰T细胞, 靶标, 毒性, Lung neoplasms, Chimeric antigen receptor modified T cells, Target, Toxicity

## Abstract

伴随人们对威胁人类健康的非小细胞肺癌（non-small cell lung cancer, NSCLC）更深入的病理生理和发病机制的全新理解，NSCLC治疗已进入一个新时代。从传统以手术、放化疗为基础的治疗过渡到以个体化精准化的靶向治疗和安全性及效能更高的免疫治疗。免疫检查点抑制剂疗法已经被批准为晚期NSCLC一线或者二线的治疗方案，并且取得非同凡响的临床效果。然而，其他类型的免疫治疗在NSCLC中鲜有探索。嵌合抗原受体修饰T细胞（chimeric antigen receptor modified T cells, CAR-T cells）在治疗几种血液系统恶性肿瘤方面表现不俗。然而，其在治疗包括NSCLC在内的实体瘤患者方面却不甚理想。本综述旨在系统阐释CAR-T在NSCLC治疗中的最新进展，主要包括：CAR分子靶标选择、CAR-T功能增强及相关毒性的管理以及CAR-T治疗NSCLC的困境及展望，旨在为NSCLC的免疫治疗开拓新的视角和独特的思路，为肿瘤免疫治疗大厦添砖加瓦。

## Abstract (en)

With the deeper understanding of the pathophysiology and pathogenesis of non-small cell lung cancer (NSCLC) which threatens human health, NSCLC treatment has entered a new era. Transition from traditional treatment based on surgery, radiotherapy and chemotherapy to individualized and precise targeted therapy and safer and more effective immunotherapy. Immune checkpoint inhibitor therapy has been approved as a first-line or second-line treatment for advanced NSCLC, and has achieved extraordinary clinical results. Meanwhile, other types of immunotherapy are rarely explored in NSCLC. Chimeric antigen receptor modified T cells (CAR-T cells) perform well in treating several hematological malignancies. However, it is not ideal for treating patients with solid tumors including NSCLC. This review aims to systematically explain the latest progress of CAR-T in the treatment of NSCLC, mainly including: CAR molecular target selection, CAR-T function enhancement and related toxicity management, as well as the difficulties and prospects of CAR-T treatment of NSCLC. It aims to open up new perspectives and unique ideas for the immunotherapy of NSCLC, and contribute to the building of tumor immunotherapy.

## 1 介绍

### 1.1 非小细胞肺癌（non-small cell lung cancer, NSCLC）免疫治疗背景

癌症免疫治疗是指利用机体自身免疫系统的力量对抗癌症的治疗策略，包括免疫检查点抑制剂疗法、过继免疫治疗以及癌症疫苗等。针对程序性死亡分子1（programmed death-1, PD1）及其配体（programmed death ligand 1, PD-L1）途径的免疫检查点抑制剂治疗晚期NSCLC表现可观^[1]^。此外，其他潜在的免疫检查点受体，如淋巴细胞活化基因3蛋白（lymphocyte-activation gene 3, LAG3）、T细胞免疫球蛋白黏蛋白结构域3（T cell immunoglobulin domain and mucin domain 3, TIM3）和杀伤性免疫球蛋白样受体（killer immunologublin receptor, KIR）等相关研究也如火如荼的开展^[2]^。过继性细胞免疫治疗是指向肿瘤患者移注在体外扩增和活化的免疫细胞，进而通过直接杀伤肿瘤或激发机体免疫反应来杀伤肿瘤细胞，达到治疗癌症的目的。

### 1.2 CAR-T细胞疗法治疗NSCLC

嵌合抗原受体修饰T细胞（Chimeric antigen receptor modified T cells, CAR-T cells）疗法是发展最为迅速的一种过继性细胞免疫治疗。该疗法是用基因工程合成受体转导患者T细胞以靶向癌细胞表面抗原，以介导抗肿瘤作用。CAR-T细胞已经被商业批准用于治疗白血病和淋巴瘤。在白血病和淋巴瘤患者中，如Tisagenlecleucel和axicabtagene ciloleucel这两种已批准的靶向CD19的嵌合抗原受体CAR-T细胞产品在造血系统恶性肿瘤的治疗中取得了显著的疗效^[3-5]^。如抗CD19 CAR-T细胞对B细胞急性淋巴细胞白血病（B lineage acute lymphoblastic leukemia, B-ALL）的治疗完全缓解率达到了90%^[6]^，这激发了越来越多的临床试验探索针对NSCLC在内的实体瘤的CAR-T细胞疗法（图 1）。

**1:**
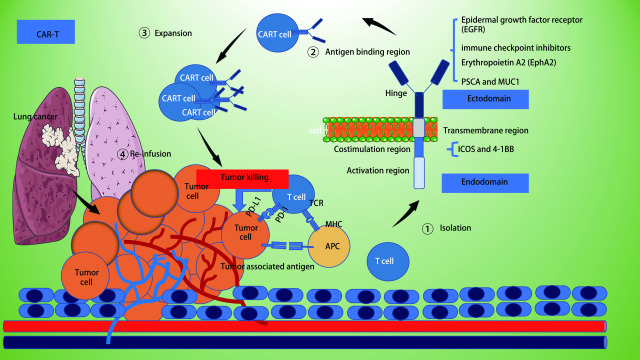
CAR-T免疫疗法治疗NSCLC。CAR-T细胞疗法的主要步骤包括：①分离体内的T细胞；②嵌合抗原受体修饰T细胞（嵌合抗原受体CAR主要包括胞内激活区和共刺激区域、跨膜区、胞外的铰链区和抗原结合区）；③CAR-T细胞的体外扩增；④CAR-T细胞回输至体内杀伤肿瘤细胞。 CAR-T immunotherapy for NSCLC. The main steps of CAR-T cell therapy include: (1) isolating T cells *in vivo*; (2) modifying T cells with chimeric antigen receptor (CAR mainly includes intracellular activation area and co-stimulation area; transmembrane area; extracellular hinge area and antigen binding area); (3) expansion of CAR-T cells *in vitro*; (4) reinfusion of CAR-T cells to kill tumor cells *in vivo*. CAR-T: Chimeric antigen receptor modified T; NSCLC: non-small cell lung cancer; APC: antigen presenting cell; MHC: major histocompatibility complex.

## 2 CAR-T治疗NSCLC的靶标选择

CAR-T在包括NSCLC在内的实体瘤要实现良好的成果转化，合理选择和利用靶标是至关重要的第一步。良好的靶标应该具有较广的覆盖率和较强的特异性以及固定的表达。覆盖率决定了CAR-T治疗的上限，而特异性则会影响治疗强度，靶标丢失将导致CAR-T治疗失败。由于实体瘤的治疗很难获得如CD19类似的理想靶标，因此，CAR-T疗法的作用不应仅限于直接杀死癌细胞，还有例如激活内源性肿瘤免疫反应和破坏肿瘤的生长环境等策略有待开发^[7, 8]^。

### 2.1 靶向表皮生长因子受体（Epidermal growth factor receptor, EGFR）

临床上治疗已经开发出多种对NSCLC靶向治疗的药物。在一项Ⅰ期临床研究中，针对复发/难治性，经基因检测表皮生长因子受体（*EGFR*）阳性（> 50%表达）的NSCLC患者，在接受剂量递增的靶向EGFR的EGFR-CAR-T细胞输注治疗后，在病理活检患者中观察到病理缓解，而且在肿瘤浸润性T细胞中检测到的CAR-EGFR基因。这表明EGFR-CAR-T细胞疗法治疗EGFR阳性晚期复发/难治性NSCLC是安全可行的^[9]^。还有研究通过优化非病毒的piggyBac转座子系统，通过工程化人类T细胞以表达EGFR-CAR。修饰的CAR T细胞在体外具有扩增能力和抗癌作用，以及在异种移植EGFR阳性肺癌患者肿瘤细胞的动物体内引起肿瘤消退^[10]^。

除此之外，EGFR变体Ⅲ（EGFRvⅢ）是一种在NSCLC中检测到的突变率为10%的肿瘤特异性突变。EGFRvⅢ-CART通过表达和释放包括穿孔素、颗粒酶B、干扰素γ（Interferon, IFN-γ）和肿瘤坏死因子α（Tumor necrosis factor, TNF-α）在内的细胞因子，特异且有效地识别和杀死EGFRvⅢ细胞。对体内和体外表达EGFRvⅢ的肺癌细胞显示出良好的抗肿瘤活性，这标志着靶向EGFRvⅢ的CAR-T是预防肺癌术后复发和转移的不可小觑的治疗策略^[11]^。

### 2.2 靶向免疫检查点CAR-T细胞

#### 2.2.1 靶向CAR-T细胞中PD-1受体

CAR-T疗法对于NSCLC的治疗效果不理想与免疫抑制性肿瘤微环境有关。NSCLC中PD-L1的表达抑制CAR-T细胞的功效。CAR-T细胞与检查点封锁相结合的组合免疫疗法策略是一种有前途的NSCLC治疗方法。除PD-1外，在肿瘤微环境还存在其他免疫抑制机制，如T细胞上还表达了多种共抑制受体（例如TIM-3、LAG3和TIGIT）。因此同时靶向多种抑制途径可能会进一步增强CAR-T细胞效力^[12]^。

#### 2.2.2 通过工程化嵌合抗原受体修饰的T细胞以分泌检查点抑制剂来增强癌症免疫疗法

有研究^[13]^设计了能分泌检查点抑制剂的CAR-T细胞（CAR.αPD1-T），并评估了它们在人肺癌异种移植小鼠模型中的功效。CAR.αPD1-T细胞具有组成性抗PD-1分泌，在体外和体内异种移植小鼠模型中，通过评价抗原特异性刺激后CAR.αPD1-T细胞的效应功能和体外扩增能力，包括IFNγ和T细胞增殖。确定了CAR.αPD1-T细胞分泌的人类抗PD-1检查点抑制剂与PD-1有效结合，并逆转了PD-1/PD-L1相互作用对T细胞功能的抑制作用，抗PD-1的分泌增强了CAR-T细胞的抗肿瘤活性并延长了总生存期。

### 2.3 NSCLC的其他靶标

#### 2.3.1 促红细胞生成素A2（Erythropoietin A2, EphA2）

EphA2在90%以上的NSCLC中过表达。因此它是NSCLC中CAR-T治疗的潜在重要肿瘤抗原靶标。具有针对EphA2的共刺激受体4-1BB的第二代CAR，在EphA2阳性NSCLC的异种移植小鼠模型中评估了体内效果，EphA2-CAR-T细胞可通过产生细胞因子IFN-γ引起肿瘤细胞裂解。用EphA2-CAR-T细胞治疗的小鼠的肿瘤信号呈下降趋势，并且比未转导的T细胞治疗的小鼠要低得多。这些结果表明EphA2-CAR-T细胞免疫疗法可能是治疗EphA2阳性NSCLC的一种策略^[14]^。

#### 2.3.2 PSCA和MUC1

前列腺干细胞抗原（prostate stem cell antigen, PSCA）和黏蛋白1（muein1, MUC1）在NSCLC中的表达。有研究结果表明，在人类NSCLC的患者异种移植（patient-derived xenograft, PDX）保持原发性肿瘤的抗原特性小鼠模型中，靶向PSCA的CAR-T细胞可以抑制PDX小鼠中NSCLC肿瘤的生长，除此之外，与靶向MUC1的CAR-T细胞联合使用甚至可以协同消除PSCA^+^MUC1^+^肿瘤。PSCA和MUC1都是NSCLC中有希望的CAR-T细胞靶标，并且这些抗原的组合靶向可以进一步增强CAR-T细胞的抗肿瘤功效^[15]^。

#### 2.3.3 靶向间皮素（mesothelin, MSLN）

的MSLN-CAR-T细胞MSLN在NSCLC中较正常组织表达丰富。针对MSLN的第二代结合了共刺激CD28和4-1BB信号域以增强增殖的CAR-T，在NSCLC中的研究表明，效应子与靶标的比率 > 0.5:1时，MSLN-CAR-T细胞比T细胞具有更强的抗肿瘤能力。另外在体内实验中还观察到，向尾静脉注射MSLN-CAR-T细胞的小鼠的肿瘤生长明显较慢。但是如果不连续给药，两组小鼠肿瘤生长情况逐渐趋同，这也显示CAR-T细胞的持久性问题有待进一步研究^[16]^。

## 3 增强CAR-T的功能及相关毒性的管理

### 3.1 增强CAR-T的功能

#### 3.1.1 ICOS和4-1BB共刺激提高CAR-T细胞的持久性

CAR-T细胞作用的持久性与肿瘤的疗效成正相关。诱导型T细胞共刺激物（inducible co-stimulator, ICOS）与Th1、Th2和Th17免疫的诱导和调节相关^[17]^。4-1BB是在活化的T细胞和自然杀伤（natural killer cell, NK）细胞上表达的诱导型共刺激受体，4-1BB在T细胞活化，持久性和记忆力中发挥重要作用^[18]^。研究^[19]^发现，CD8^+^ T细胞的持久性依赖于用于重定向CD4^+^ T细胞的细胞内信号传导域（intracellular domain, ICD）提供的辅助作用。与基于4-1BB的CAR相比，在实体瘤模型中第三代CAR-T cells中结合使用ICOS和4-1BB ICD表现出优异的抗肿瘤作用并提高了体内持久性。

#### 3.1.2 细胞外模块（铰链结构域）对CAR-T治疗效果的影响

CAR的更新迭代，主要集中在细胞内信号传导模块上。无信号的细胞外模块对CARs的扩增和治疗效果研究甚少。有研究通过生成两个带有或不带有铰链域的CAR载体（分别靶向CD19、间皮素、PSCA、MUC1和HER2），系统地比较了铰链域在体外和体内对CAR-T细胞生长动力学，细胞因子产生和细胞毒性的影响。体外迁移试验表明，铰链增强了CAR-T细胞的迁移能力，证明铰链有助于CAR-T细胞扩增。有趣的是，抗CD19 CAR的T细胞在体内具有抗肿瘤能力与是否含有铰链无关，而表达包含铰链域的抗间皮素CAR的T细胞显示出增强的抗肿瘤活性，证明铰链能够提高某些特定CAR-T细胞的抗肿瘤功效，需要更多的研究来进一步探索^[20]^。

### 3.2 CAR-T细胞学治疗毒性

CAR-T细胞疗法使用的相关不良事件，主要是细胞因子释放综合征（cytokine release syndrome, CRS）和神经系统毒性。其中CRS的表现包括血细胞减少、凝血病和吞噬性淋巴细胞组织细胞增生等。神经系统毒性包括癫痫发作和脑水肿等多种临床症状。CAR-T细胞剂量和CAR设计与其不良反应相关。在研发改良的CAR设计和T细胞产生方法可以改善全身持久性和活性同时，也需要对CART细胞毒性进行进一步的临床研究^[21, 22]^，虽然大多数不良事件可分为轻度或中度，但也会发生需要入ICU治疗的严重且危及生命的并发症。在实施细胞疗法抗肿瘤药物治疗后的重症癌症患者，应在ICU工作人员、肿瘤学家和器官专家的团队配合下进行管理。同时需要更多临床和临床前研究改进相关不良事件的病理生理学认识及其诊断和治疗管理^[23]^。

## 4 总结

CD19CAR-T在血液系统恶性肿瘤取得非凡成就，与其B细胞谱系细胞上的CD19统一且特异性表达关系密切。迄今为止，NSCLC等实体瘤中的肿瘤相关抗原（tumor-associated antigen, TAA）靶标在强度和分布方面表现出异质性，缺乏真正的肿瘤特异性抗原，这极大的限制了CAR-T的临床效果。研究新颖的CAR设计克服抗原异质性势在必行，例如靶向双TAA的CAR、串联CAR和可转换CAR以及抑制性CAR^[24]^。CAR-T疗法最佳临床效果，包括CAR-T细胞的活化、持久性和较小的CAR-T细胞介导的毒性。有必要将多种修饰结合起来，不断优化CAR-T细胞的亲和力和持久性^[25]^。值得关注的是，NSCLC作为实体瘤物理障碍也亟需解决，研究开发标准化的3D肿瘤模型，使药物在临床应用前获得其安全性和有效性的验证^[26]^。应进一步透彻了解CAR不同结构域的功能以及相互影响，不断改善CAR-T细胞的设计，以取得更好的治疗效果。根据不同的免疫治疗策略，为NSCLC患者免疫治疗提供更多的选择和方案。
